# Cutting edge strategies for screening of novel anti-HIV drug candidates against HIV infection: A concise overview of cell based assays

**DOI:** 10.1016/j.heliyon.2023.e16027

**Published:** 2023-05-05

**Authors:** Shraddha Y. Gaikwad, Pallavi Phatak, Anupam Mukherjee

**Affiliations:** Division of Virology, ICMR-National AIDS Research Institute, Pune, MH, India

**Keywords:** HIV, Cell based assays, Drug discovery, Preclinical screening, Antiretrovirals

## Abstract

The advent of Highly Active Antiretroviral Therapy has majorly contributed towards reducing the morbidity and mortality associated with HIV infected people, thus improving the quality of their life. Still, the eradication of HIV infection has not been achieved due to some important limitations such as non-adherence to therapy, cellular toxicity, restricted bioavailability of antiretroviral drugs and emergence of drug resistant viruses. Moreover, persistence of latent HIV-reservoirs even under antiviral-drug pressure is the major obstacle in HIV cure. Currently used antiretrovirals can suppress the viral replication in activated CD4^+^ cells, however, it has been observed that the available antiretroviral therapy appears inadequate to reduce latent reservoirs established in resting memory CD4^+^ T cells. Therefore, for eradication or reduction of latent reservoirs many immunotherapeutic and pharmacologic approaches including latency reversing agents are being studied constantly. Additionally, promising therapeutic strategies including discovery of novel drugs and drug targets are continuously being explored. Therefore, preclinical testing has become an important step of drug development process, continuously demanding innovative, but less time consuming evaluation strategies. Present review attempts to gather and line-up the information on existing cell-based methodologies applied for assessing drug candidates for their antiretroviral potential. Further, we intend to outline the advanced and reliable cell based methodologies that would expedite the process of discovery and development of antiretrovirals.

## 1 Introduction

In view of the significant limitations of present Highly Active Antiretroviral Therapy (HAART), discovering, developing, and repurposing antiretroviral agents have been a constant need of recent times [1]. Therefore, chemical entities, herbal and bio-active products including peptides, therapeutic antibodies and biomolecules have been used as therapeutic agents. Moreover, to overcome the hurdles in the present therapeutic interventions, the researchers are in surge of developing strategies for the discovery of new drug-like products, novel drug targets, and innovative drug delivery systems. The journey of each novel antiretroviral agent starts in the laboratory and ends at the pharmacy counter after many turns and stretches. An integral part of this journey is preclinical testing involving *in vitro* and *in vivo* experiments [2]. *In vitro* preclinical testing provides information on the safety and efficacy of a new drug, requiring appropriate testing platform, to generate accurate, reliable, and reproducible results [2]. After exhibiting the safety and efficacy potential, at the preclinical testing stage, only the promising drug candidates can be taken up for further studies in animals and humans [2]. Given this, the preclinical testing becomes an important step for deciding the fate of a new drug candidate. Hence, for evaluating a large number of drug candidates in a cost and time-effective way researchers have always thrived for innovative, promising and simple methodologies. Among them, cell based experiments have found to be the most reliable approach, therefore, having major contribution in the discovery of presently available antiretrovirals.

The current review summarizes the presently employed cell-based methodologies adopted for the evaluation of antiretroviral drug candidates. It also attempts to outline the futuristic approaches that will support accelerating the process of discovery and development of more powerful antiretrovirals (ARVs).

### 1.1 Antiretroviral drug discovery and development

Broadly, the antiretroviral drugs have been classified into two groups, direct-acting strategies and host factor-targeting therapies [3]. The direct-acting group targets the virus directly at each step of its replication cycle like reverse transcriptase, integrase and protease inhibitors interfere with the virus directly and interact with enzymes, essential for viral replication. The host factor-antiretrovirals target the cellular receptors, for example, antibodies binding to CD4 receptors and CCR5/CXCR4 co-receptors required for viral infection and pathogenesis [3]. For years, so many chemical compounds, herbal and marine products, biomolecules, small peptides, antibodies, nanomedicines and RNA based therapeutics have transformed into antiretroviral drugs, passing through the stages of clinical trials. Further expansions in the field of drug discovery and pharmaceutical technologies, HIV therapeutics have progressed towards the development of sustained release drugs, nanoformulations and nanocarrier systems, RNA therapeutics and targeted delivery models, therapeutics for eradication of latent HIV-reservoirs, generating broadly neutralizing antibodies (bNAbs) and therapeutic vaccines [3]. The modern experimental systems and high throughput screening (HTS) platforms have encouraged the assessment of wide range of drug candidates, right from the preclinical stage itself. Besides, safety and efficacy, much more information about drug-cell, drug-biomolecule interactions, etc. have been provided by various cell based systems [2]. Therefore, the present review attempts to describe *in vitro* cell based experiments, currently available for testing the activity of therapeutic agents against HIV.

### 1.2 *In vitro* screening: biochemical & cell based experiments

For years, the *anti*-HIV drug candidates have been tested by both approaches, biochemical as well as cell-based assays, also known as bio-assays. Biochemical assays are target-specific where, targeting either HIV-1 reverse transcriptase or protease enzyme, one at a time. The prerequisite of these experiments is the detailed knowledge of target molecules, which makes them suitable for known classes of inhibitors [4]. Moreover, they may become challenging if the protein target is difficult to purify [4]. Importantly, biochemical screening lacks the complete set of physiological conditions, making them less efficient in predicting the clinical value compared to cell based experiments [4].

**Fig. 1:**
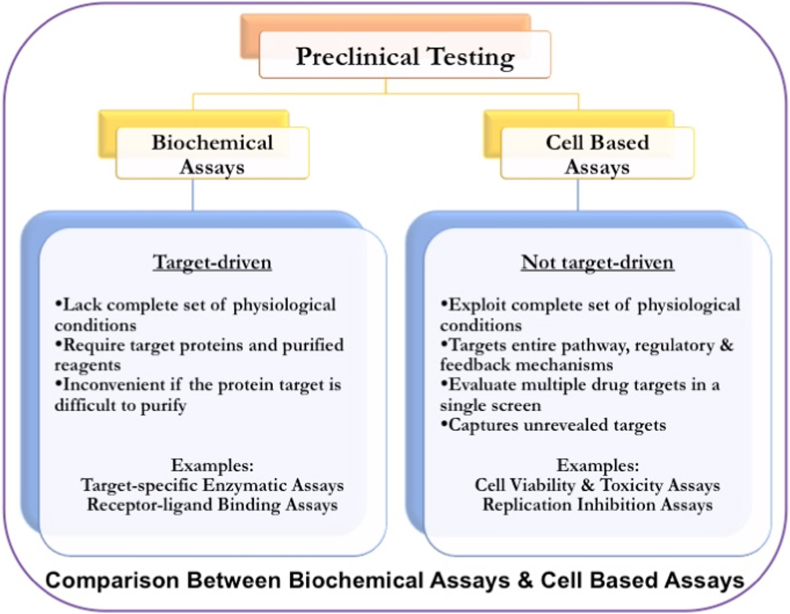
Strategies for Preclinical Testing of antiretroviral drug candidates - The Biochemical Assays are target driven and lack of complete set of physiological conditions, whereas, the Cell Based Assays are not target driven, exploit complete set of physiological conditions.

On the other hand, cell-based assays are more in agreement with a complete set of physiological conditions and are not target-driven [4]. Here, entire biological pathways with complete regulatory and feedback mechanisms are considered to be the target. Moreover, cell-based systems can evaluate multiple drug targets in a single screening process, and they can capture unrevealed targets that are missed from biochemical screening [4]. Additionally, cellular context provides information on membrane permeability and toxic effects on the host cells. Unlike biochemical assays, cell-based assays allow the screening of compound libraries for cytotoxicity and antiviral activity in intact cells at an early stage of the drug discovery and development process [4]. This approach, being closer to the biological system, has progressed rapidly with biotechnological support, generating reliable and reproducible results greatly influencing the drug discovery and development campaigns (Fig. 1).

## 2 Cytotoxicity testing

*In vitro* cytotoxicity testing of drugs is important at the early preclinical stage which decides the fate of a drug candidate in the journey of clinical research. Cytotoxicity screening quantitatively measures the effect of any drug candidates on the cell viability, cell growth and simply enumerates the cells upon exposure of drug candidates [5,6]. It is usually determined before commencing the efficacy studies on antiretrovirals. The basic principle of cytotoxicity experiments is based on the ability of the cell to reduce compounds, such as 3-(4,5-dimethylthiazol-2-yl)-2,5-diphenyl-2H-tetrazolium bromide (MTT), 2,3-bis (2-methoxy-4-nitro-5-sulfophenyl)- 5-[(phenylamino) carbonyl] -2H-tetrazolium hydroxide (XTT) in presence of candidate drugs. Common experimental platforms have been established based on these reactions. In addition, the Neutral Red Uptake (NRU), Resazurin reduction (RES) and sulforhodamine B (SRB) assays have also been used for cell enumeration [6].

The MTT assay, being the popular and the gold standard method [6,7], has been described here. It is a colorimetric assay that measures the enzymatic activity of cellular oxidoreductase and dehydrogenases enzyme present respectively in the endoplasmic reticulum and mitochondria of living cells. These enzymes reduce the tetrazolium compound-MTT into its water-insoluble, purple colour formazan crystals. Here, the formazan crystals formation is influenced by the metabolic rate and number of mitochondria present in the cell [5]. These water-insoluble crystals are then dissolved in dimethyl sulfoxide (DMSO) and absorbance of coloured product is measured by a spectrophotometer. Thus, absorbance is directly proportional to the cell viability, indirectly indicating the level of cellular toxicity. Given this, the dose dependent toxicity of an investigational drug after exposure to the cells, for specified time interval is determined and compared with the appropriate drug control with known toxicity profile. The results are expressed in terms of CC_50_ value, defining the concentration of drug candidate at which at least 50% of cells are viable [8]. Thus CC_50_ value becomes a measure of cellular toxicity and safety which further directs several viral infectivity assays. Likewise, the cytotoxicity screening is performed in-parallel to the infectivity assays to differentiate between the viral inhibition achieved by candidate's activity or by cell death, due to drug's toxicity or distressed atmospheric conditions. Even though the MTT assay is simple to perform and known to be the gold standard method, it has a few limitations pertaining to its applications in cytotoxicity studies. The point of concern is deposition of the needle-shaped MTT formazan crystals that injure cell membranes and disrupt cell integrity by activating apoptosis related factors such as caspase-8, caspase-3 or accelerate the leakage of cell contents is the point of concern [7]. Moreover, it has been reported that MTT assay interferes with certain compounds like glycolysis inhibitors that affect cell metabolism. It also interacts with many phytochemicals demonstrating intrinsic reductive potential including antioxidants and polyphenols [6]. Such reports reopen the discussion on reliability of MTT assay in cellular toxicity studies.

The Neutral Red Assay (3-amino-7-dimethylamino-2-methylphenazine hydrochloride) is a measure of viable cells ability to incorporate and bind to supravital neutral red dye. The lysosomal uptake of neutral red depends on the cells’ capacity to maintain pH gradients, through the production of ATP and it is independent of enzymatic conversion of dye [5,6]. Though the method is sensitive, certain volatile and unstable compounds have presented interferences [5,6]. On the other hand, Resazurin assay (RES), which is also known as Alamar blue assay, measures the metabolic activity of a living cell. The mitochondrial reductase enzyme occurs mostly in the mitochondria, and converts the nonfluorescent dye Resazurin to the strongly-fluorescent dye Resorufin that can be monitored using a standard spectrophotometer [5]. Thus, the quantity of Resorufin is a measure of metabolic activity of a cell. The assay is simple rapid and more sensitive than tetrazolium based assays [6]. The main advantage of Alamar blue is its non-toxic nature, which allows the return of exposed cells to the culture flask for performing any follow-up assays [5,6]. The Sulforhodamine B (SRB) assay is also based on binding of a fluorescent dye to the basic amino acids of cellular proteins. Here, the total protein mass related to the cell numbers, can be evaluated colorimetrically. The advantages include sole dependency on protein content, higher sensitivity and reproducibility, better linearity, and no compound interference [6]. Nevertheless, the assay sensitivity reduces with the nonadherent cells. Hence, it has been concluded that the MTT assay possesses largest variation in linear range compared to the above commonly used cell enumeration assays [6]. Overall, it indicates the vigilant selection of MTT assay for evaluating the cellular viability and/or toxicity.

Another promising approach for monitoring the effect of drug candidates on cells is determination of the level of Adenosine Triphosphate (ATP). As ATP is present in all metabolically active cells, plays an essential role in the energy exchange, hence recognized as a marker of the functional integrity of a cell. This attribute makes ATP measurement essential while studying the living processes. Basic principle of this method is catalytic reaction between enzyme Luciferase, obtained from firefly, *Photinus pyrali*s and substrate luciferin [9,10]. The method has high specificity because only the viable cells can provide ATPs required for the reaction [9,10]. The reaction proceeds with two steps, first, the luciferin is activated to give luciferyl-adenylate and pyrophosphate, followed by the reaction between luciferyl-adenylate and molecular oxygen, yielding oxyluciferin. When excited-oxyluciferin returns to the ground state, green to yellow luminescent light is emitted and its intensity is then measured using a light sensitive apparatus, Luminometer. The readouts are expressed in terms of Relative Luminescence Units (RLU) that are proportional to concentration of ATP in a cell [9,10]. The experimental data is analysed similar to MTT assay. It has been reported that the assay is less prone to artefacts, compared to the other traditional cell viability assays and is sensitive to detect signals from the minimal number of cells. Moreover, to make the assay compatible with HTS platforms, it has been modified to generate a stable luminescent signals that can tolerate harsh cell lysis conditions and are resistant to luciferase inhibitors found in small molecule libraries. Appreciating the sensitivity, specificity and simplicity of these ATP determination assays, they have been widely utilized in preclinical studies.

### 2.1 Reporter gene assays

**Fig. 2:**
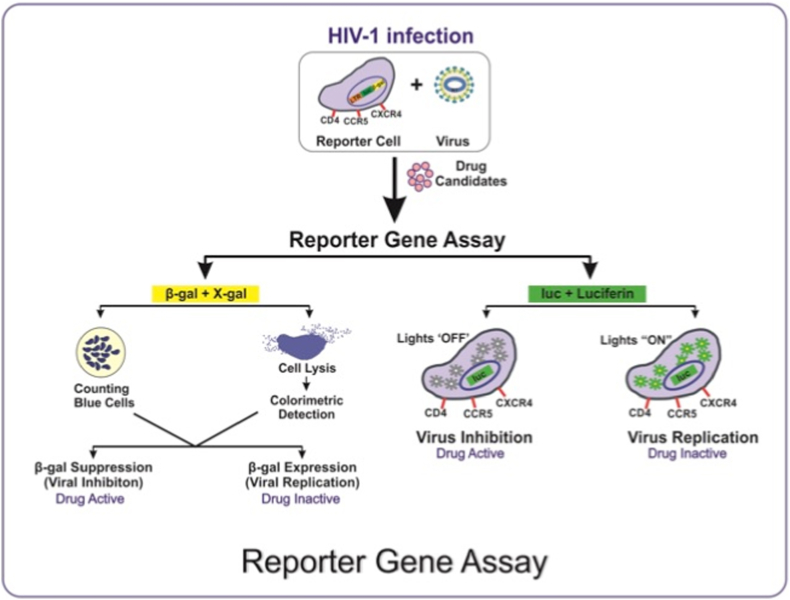
Principle of Reporter Gene Assay - The basic principle of this assay is based on the incorporation of reporter gene into the cell; Higher expression of reporter gene indicates active viral replication, whereas, the suppression of reporter gene expression indicates inhibition of viral propagation for measuring the potential of any drug candidates.

The antiretroviral research is incomplete without the reporter gene assays. These experiments are based on expression of reporter genes that can indicate the activation of a certain gene, articulated in a biological system such as bacteria, viruses, and human cells. In antiretroviral studies, to measure the extent of viral replication, reporter genes have received much attention. Few important reporter genes include: (**1**) Green fluorescent protein (GFP) expressing cell glow in green fluorescence under blue light, (**2**) Luciferase enzyme emitting luminescence upon catalytic reaction with luciferin, and (**3**) Beta-galactosidase enzyme reacts with X-gal (chromogenic substrate) producing a blue product [[11], [12], [13]]. Among these, the luciferase gene assay system has become the most reliable assay because of its high sensitivity, reproducibility and minimal background luminescence in most of the cell types [14]. As described earlier, the reaction of luciferase with substrate luciferin emits photons of light that can be detected by luminometer. Here, the expression of luciferase gene indicates HIV-1 replication, which is regulated by HIV-1 LTR (Long Terminal Repeats) promoter. In contrast, non-expression of reporter gene indicates virus neutralization due to the action of a drug candidate or by an antibody [14]. Based on this concept, various cell based experimental platforms have been developed and practiced for monitoring the replication and inhibition of HIV infection (Fig. 2). Among these systems, TZM-bl/Luciferase gene assay system has demonstrated its fidelity and reproducibility for screening antiretroviral drug candidates. The TZM-bl cell line is adherent, monolayered, expresses high levels of CD4 receptor and CXCR4 & CCR5 co-receptors and hence, permissive to molecular clones and primary isolates of HIV-1 variants. Also, it has been genetically engineered with two reporter genes: Firefly luciferase and Beta-galactosidase. The reporter gene expression is induced in trans by viral Tat protein under the control of HIV-1-LTR (Long Terminal Repeats) [[11], [12], [13]]. Thus, reduction in HIV-1-Tat-regulated luciferase (Luc) reporter gene expression is a function of viral neutralization [11]. To date, the TZM-bl assay platform is being utilized for screening antiretroviral properties of large number of novel drug candidates [8,14,15]. Furthermore, it has been reported that the growth medium should be supplemented with DEAE-Dextran at a concentration of 20–40 μg/ml for optimal HIV-1 infection [12]. However, the LTR-driven transcription of HIV-1 reporter via TZM-bl cells is less efficient to record the late replication events such as production or packaging of viral particles [16]. Nevertheless, TZM-bl cells have been used as cellular model for HIV latency and transcription mechanistic studies [17].

The assay protocol for screening antiretroviral drug candidate comprises cytotoxicity testing followed by the testing of the inhibitory potential of subtoxic concentration of a drug candidate against HIV-1 [8,15]. However, prerequisite of designing these antiviral experiments is assurance or presumption of mechanism of action (MoA) of a drug candidate. With the progresses in computational analysis, certain in silico studies have been developed for prediction of MoA. It includes databases, quantitative structure-activity relationships, pharmacophores, homology models and many more. *In silico* methods thus help in designing further *in vitro* experiments. Overall, the cell based experiments have been designed according to the MoA of an investigational drug.

**Fig. 3:**
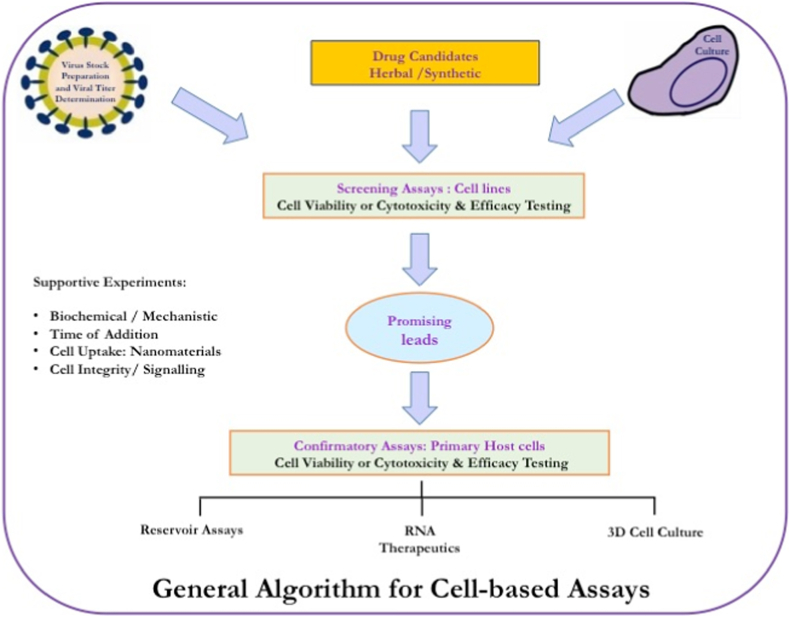
Illustration of general procedure for Cell based Assays - The algorithm comprises a set of experiments, initiates with screening of drug candidates using multiple cell types, followed by Confirmatory Assays and other mechanistic assays *viz.* (i) Biochemical Assay, (ii) Time Addition Assay, (iii) Cell Uptake Assay for nanomaterial or nano-carrier based targeted delivery, (iv) Cell Integrity Assay for pathway specific signalling study.

Increasing knowledge on HIV-1 replication steps and cellular host factors contributing in viral replication has offered several drug targets which then resulted in various forms of TZM-bl experiments. Thus, multifaceted experimental set up, reliable and reproducible results have received the attention of many researchers in this field. Here we describe the overall protocol for antiretroviral screening of an investigational drug, showing probable MoA as HIV-1-replication and/or reverse transcriptase inhibitor through cell-based assay (Fig. 3). The pre-seeded cells are infected by HIV-1 for around 2 h and then exposed to sub-toxic concentrations of a drug candidate/drug control, followed by 48hr incubation and luciferase gene assay [8,18]. Similarly, for evaluating virucidal agents, a drug-treated virus is allowed to infect the pre-seeded TZM-bl cells [15]. In this way upon extensive research and considering various HIV-replication steps, many experimental recipes involving different combinations of treatment of cell or virus with the drug have been developed. Finally, these experiments have aimed at monitoring the extent of viral replication or inhibition under drug pressure. Importantly, the measurement of luciferase reporter gene expression has been a universal termination assay for all such experiments, making the interpretation and comparison of results easier. The RLUs from virus infected-drug-treated cells are compared with virus-infected-untreated cells, commonly designated as virus control (VC) and uninfected-untreated cells serves as cell control (CC), expecting, median, maximum and minimum expression of RLUs, respectively. Hence, the virus replication is measured in terms of RLUs. At the end, the dose-dependent inhibition pattern, the concentration of a drug showing 50% inhibition of virus (IC_50_ value) and the ratio of CC_50_ to IC_50_ value, known as selectivity (SI) or therapeutic index (TI) of a drug candidate is determined and compared with drug standards [8]. A drug candidate showing a comparable inhibition pattern or IC_50_ value with its respective drug control is recognized as a promising lead. Thus, the TZM-bl/Luciferase gene assay system has become the popular technology due to its simplicity, sensitivity, reproducibility and compatibility with low and high throughput systems.

Alternative approach for screening antiretrovirals has also been explored by targeting the other reporter gene, encoding β-galactosidase enzyme. The gene, incorporated in HeLa-CD4-LTR-β-gal cells gets activated upon HIV infection [19], and the β-galactosidase enzyme when reacts with its substrate X-gal produces blue coloured product. Enumeration of the blue stained cells or measurement the intensity of blue coloured product is proportional to viral replication. For quantification purpose, two methods have been utilized, counting of blue stained cells and colorimetric detection of enzyme-substrate reaction. The comparative study of these methods has shown good agreement between them [19]. Nevertheless, the labour intensive counting of the blue stained cells has restricted its application.

Later on, the comparative analysis between luciferase and β-galactosidase reporter gene assays have confirmed the superiority of luciferase gene assay owing to its reliable and reproducible results [13]. Together, it can be concluded that, reporter gene assays, especially luciferase gene assays have been the most useful tool for screening various antiretrovirals and to study diverse characteristic interactions between drugs, cells and viruses.

### 2.2 Replication inhibition assays

Although reporter gene assays have proved their suitability for screening novel antiretrovirals, afterwards, they have found to be laborious and time-consuming. Moreover, separate experimental setup is needed for evaluation of every class of new drugs. Hence, to reduce the time and efforts, a strategy pointing towards the entire HIV-1 replication cycle has been developed wherein a complete set of drug target has covered. The experiment based on this strategy has been recognized as HIV-1 full replication assay (Rep assay) [20]. In the following years, the same strategy has been used to establish a reliable, reproducible and full proof screening platform, namely, Exploratory Assay System for the discovery of HIV Inhibitors *i.e.* EASY HIT technology [16].

The strategy involving HIV-1 Rep Assay has adopted two different cell lines, for monitoring the HIV-1 replication. For initial infection HIV-1 permissive, MT-2/PM1-T cell lines and to demonstrate HIV infection, HeLa CD4 LTR/β-Gal indicator cells have been used [20]. Here, virus replication is measured in terms of HIV-1 tat induced β-Gal activity using a dual-light system and a Microbeta Luminometer system. The reporter activity in infected, non-infected and infected-compound-treated cells has been compared and expressed as percentage inhibition. Compounds showing ≥50% inhibition were identified as Hits. After performing validation of reporter gene signals, signal-to-background ratios, and intra assay variability, the MT-2 cell-based system has proved its suitability to the HTS platform, attributed to lower intra assay variability, lower false negativity and better reproducibility. Interestingly, the HIV-1 Rep assay system has proven advantageous for late-phase inhibitors [20]. Additionally, the HIV-1 Rep assay has shown that T-cell component and functional vif gene is required for high levels of viral replication and reporter gene expression. Given this, it can be concluded that, the replication inhibition assay offers a full proof, simple and flexible screening platform. Prospectively, the idea of targeting entire replication cycle for screening of HIV-1 inhibitors would identify the early and late phase inhibitors at the initial stage of drug development process itself, expediting the method.

### 2.3 EASY-HIT system

**Fig. 4:**
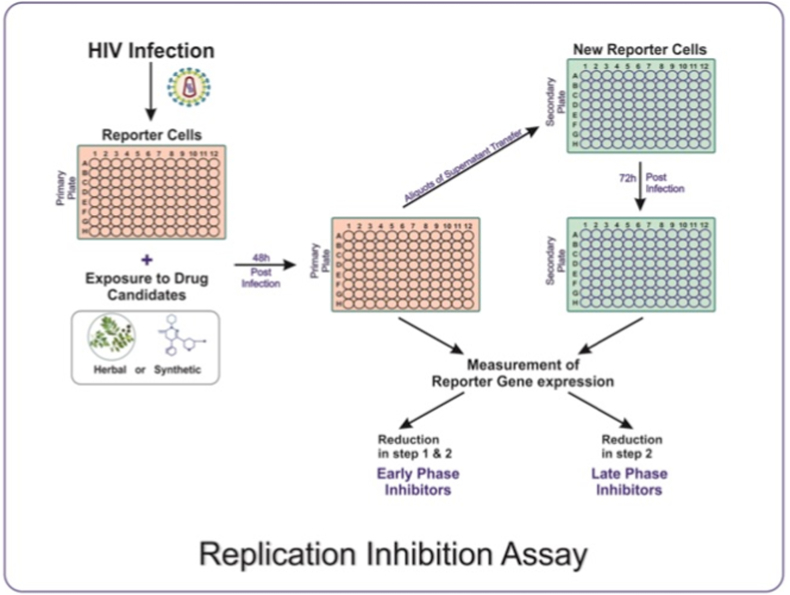
Repesentation of basic procedure for Replication Inhibition Assay **-** The experiment assess the complete set of drug targets in the HIV-1 replication cycle. Reporter cell-lines are exploited to group the drug candidates into early and late phase inhibitors.

The strategy of targeting full replication cycle of HIV has been refined by establishing the EASY-HIT system [16]. The system has a potential to identify and differentiate between HIV inhibitors acting on various stages of HIV replication. Earlier described screening platforms have favoured the rational drug designing approach where chemical entities have been well characterized for purity, solubility and MOA, targeting specific drug sites in HIV replication cycle. Moreover, the reporter gene systems based on activation of HIV-Tat protein under the transcriptional control of the LTR have shown background expression attributed to the inherent basal activity of HIV LTR and its responsiveness to external stimuli [16]. This approach limits their application potential and eventually increase longitivity of drug development process. On the other hand, The EASY-HIT system has presented an attractive strategy, especially for screening less studied chemicals and crude natural products. The LC5-RIC cells, where LC5 is the HeLa derived cell clone expressing CD4 & CXCR4 receptors, and RIC is Red infected cells have been a backbone of this system [21]. These cells are transfected with Rev and Tat-dependant reporter gene with red fluorescent protein (DsRed1) having good susceptibility to HIV infection. Additionally, the lack of background reporter gene expression makes the system more accurate [16]. The system comprises two-step assay protocol, enabling simultaneous identification and classification of HIV inhibitors into early and late phase inhibitors of HIV-1 replication (Fig. 4).

Typically, the experiment is carried out on preseeded LC5-RIC reporter cells that are exposed to candidate inhibitors. Within 30 min of treatment, the treated cells are infected by HIV-1. After 2 days post-treatment, the inhibitory effects of drug-candidates are assessed by measuring fluorescent signal intensities from primary cell cultures. Subsequently, aliquots of culture supernatants from primary cell cultures are transferred to newly seeded LC5-RIC cells, referred as secondary cell cultures [16]. After 3 days post-transfer of the supernatent, fluorescent signals are measured again. Here, reduction in relative fluorescence intensity (RFI %) is the measure of replication inhibition. Also, the experiment is performed under controlled conditions, similar to reporter gene assays. The assay system has been validated with a panel of known HIV-1 inhibitors. Given that, the system is capable of identifying the early phase inhibitors, targeting entry, reverse transcriptase, Integrase & transcription steps, and late phase inhibitors having HIV protease inhibition activity. The inhibitors showing replication inhibition in both steps, the primary and secondary cultures, have been categorised as early phase inhibitors and those inhibiting the virus predominantly in the secondary cultures have been designated as late phase inhibitors [16]. With this, it can be concluded that the EASY HIT system has provided a powerful and reliable platform for screening antiviral activities of less-studied synthetic, crude herbal products and their mixtures because it aims at full replication cycle rather than targeting one step at a time. Furthermore, enabling early/late phase-classification of HIV inhibitors at initial level of developmental process will narrow down the scope of MoA determination studies, expediting the drug discovery and development process.

### 2.4 Experiments based on time of addition

As discussed earlier, determining the probable MoA of an investigational drug has always been an important aspect of rationally designed cell based experiments. Hence, for defining the MoA, a simple and straight forward strategy called Time of Addition (TOA) or Time Point Addition (TPA) experiments have been utilized, since years [22]. Alternatively, antiretroviral agents screened by several approaches, can be confirmed for their MoA by TOA experiments. Basically, time bound sequential events in HIV replication cycle has encouraged the designing of TOA assays. These implicate exposure of virus-infected cells to the drug candidate/drug control, added at various time points. These time points have been depicted from earlier studies on viral replication events. Through these studies it have been observed that, the addition of an inhibitor before the respective replication step has halted the replication cycle. Otherwise, adding an inhibitor after a specific replication step has not the ability of terminating the viral replication. Moreover, to predict the MoA of unknown inhibitors, their loss-of-inhibition activity at certain time points is compared with their respective drug controls [23]. Thus, the time of addition of a drug candidate that ceases a specific replication step of HIV, analogous to its relevant drug control, outlines the MoA [22]. Broadly, TOA experiments are based on the highly permissive cell lines for HIV-1 such as T-cell line or TZM-bl cell line [8]. The cells are allowed to infect by HIV-1 at high multiplicity of infection (MOI) or with optimal cell culture infectivity dose (TCID_50_). To synchronize the infection, the cells are washed and exposed to subtoxic concentration of inhibitors/drug controls, 10–100 fold more than their IC_50_ value [8,22]. However, such high concentration has raised a challenge in case of drug candidates showing toxicity at 10–100 fold concentration of the IC_50_ value. Virus inhibition is then measured depending on the cell line used, either by employing ELISA for capsid protein, p24 or by checking the reporter gene expression [22]. Therefore, TOA merits in describing and discriminating the possible MoAs of inhibitors and directs future studies on drug characterization and profiling at initial phase of drug discovery itself. Further, it has been observed that TOA has good compatibility with herbal extracts demonstrating the loss-of-inhibition activity even at earlier time points compared to the drug control used [24]. The study has revealed that the tested herbal extract has entry inhibitor activity that blocks the attachment of virus particles to the cells [24].

Nonetheless, the manual-TOA has an important limitation of laborious and error-prone addition of the test products especially when the time intervals for drug additions are short [22]. Therefore, automated instrumentation platforms have been established to overcome these limitations. An instrument having liquid handler and an incubator with a rail interface, integrated with software packages with user friendly programmes has been invented. This facilitates the error-free and customizable additions at fairly shorter time intervals. Although, such automations have been found to be rapid and user-friendly, pricey liquid handler system and its softwares are the limiting factors, especially in developing countries.

#### 2.4.1 Cellular uptake

The safety and efficacy of a drug candidate is influenced by drug-cell interaction, its transportation to the target cell, intracellular absorption & concentration and excretion. Also, the Pharmacokinetic study majorly deals with drug absorption for which diverse mechanisms have been identified, including passive diffusion endocytosis, pinocytosis, phagocytosis or some receptor or carrier mediated intake [25,26]. Therapeutic agents ranging from small molecules like peptides to Drugs/Nano formulations to the large macromolecules like proteins enter the cells by these mechanisms. Of these, passive diffusion is the most common mode of entry where drug molecules move according to the concentration gradient until equilibrium is reached. While carrier mediated membrane transport utilize some essential biological processes such as energy, ion & nutrient transport systems, for the absorption of drug molecules. As most of the drugs have intracellular targets, their successful intake & internalization has always been an important concern. Accumulation or partial release of drug in cellular compartments may result in degradation of drug, rendering the treatment ineffective [25]. Hence, for better therapeutic interventions, it is essential to gauge the cellular uptake that plays a vital role in drug's bioactivity. Eventually, this would facilitate the development of more effective therapeutics. Therefore, drug-uptake assays have been adapted for deciding drug's fate inside the cell. In general, for carrying out the uptake assay, cells of interest are exposed to drugs under investigation which can be labelled using albumin coated antibody or any other fluorophore [26]. Cells treated with such conjugated drug-tag complexes are then subjected to microscopic and imaging techniques, including fluorescent and confocal microscopy, microspectrofluorometric analysis, and Raman microspectroscopy. Any of these affordable and suitable techniques have chosen for this evaluation. Earlier studies on HIV drug resistance and reservoirs have stated that, drug's effective concentration and adequate distribution at a specific site with optimal time-duration is imperative for a therapeutic efficiency [27]. Also, anatomically sequestered sites like secondary lymphoid tissue, liver, gut and brain have been recognized as the HIV-reservoirs where infiltration of drug is suboptimal which favours reactivation of virus leading to drug resistance [28,29]. Moreover, resting CD4^+^ T cells and cells of monocyte/macrophage (M/M) lineage has been an important reservoirs of HIV [30,31]. Possibly, the reservoirs formed by M/M are the cause of plasma viremia [32,33], and eventually become more resistant to apoptosis; giving rise to persistent HIV-reservoirs, finally contributing in ART resistance [34]. Therefore, for effective treatment and for preventing emergence of viral reservoirs it is essential to explore novel approaches which would improve drug delivery and bioavailability at targeted site. Employing nanomaterials for drug delivery has been a promising and well-practiced strategy. Nanomaterials have also been found effective in delivering antiretrovirals in infected cells as well as HIV reservoirs including macrophages [35,36]. The cellular uptake of nanoformulations is influenced by its physical properties like size, shape and chemical properties like, surface charge, solubility and stability [25,37]. Explicitly, their internalization and mechanism of action can be studied by using certain spectroscopic techniques such as Raman spectroscopy and confocal microscopy. These techniques can reveal the subcellular accumulation, distribution, MoA of a drug and its time of action as well. Such advance and accurate spectroscopic techniques have modernized the cell uptake assay which can strengthen the antiretroviral drug discovery and development programs. Indeed, it has also been evidenced that nanosystem can be taken up rapidly by cells such as THP-1 macrophages which are capable of delivering therapeutic agents, like siRNAs, into the cytoplasm and targeted sites by escaping lysosome or endosome compartment [37]. Therefore, the researchers have chosen the strategy of infected macrophage-nanosystem for targeting HIV-1 reservoirs. Certainly, this approach can be reciprocated to target other macrophage-proliferating pathogens such as malaria and tuberculosis.

Hence, it can be concluded that, cellular drug uptake assays would be beneficial in early pre-clinical stages to assess the ability of drug formulation and nanoparticles to cross the plasma membrane and achieve optimum intracellular concentration. Such innovative approaches highlight the need for exploring novel cell based or spectroscopic methodologies pointing at HIV reservoirs that would surely contribute in combating various infections.

#### 2.4.2 HIV-1 reservoir assay

Despite standardized and full-proof antiretroviral therapy, HIV-1 still persists in patients for years, leading to viral rebound and non-curable HIV infection. This is a major consequence of concern from the last few years. Latently infected resting CD4^+^ T cells play key role in existence of HIV reservoirs. In these cells, an integrated copy of the viral genome persists in various forms without any expression [38]. Attempts for targeting these latent reservoirs and the development of a rapid, feasible, scalable and reliable assay for quantifying these reservoirs are in a great surge. A variety of novel experiments have recently been described for accurate measurement of viral reservoirs. Quantitative viral outgrowth assays (QVOA) releasing infectious virus upon single round of T cell activation can define the viral reservoir [39]. In addition, few Latency Reversing Agents (LRAs) such as Suberoylanilide Hydroxamic acid (SAHA), also known as Vorinostat, and Inhibitor of Apoptosis Protein (IAP) antagonist (IAPi) have also been used to disrupt latent HIV with minimal immune activation [40,41].

Standard PCR technique, targeting the conserved regions of HIV-1 genome has been used commonly, but due to huge number of defective HIV-1 proviruses, the PCR could not capture minor subset of replication-competent proviruses [42]. In the past, the pioneering studies on latent reservoirs of HIV-1 demonstrated the QVOA by means of resting CD4^+^ T cells [39]. The method has been referred as “Gold Standard” for quantification of frequency of resting CD4^+^ T cells harboring latent HIV-1 [38]. Conventionally, the assay has been confined to the purification of cells obtained from the HIV infected donors under suppressive ART. The limiting dilutions of infected cells are then plated in a specified format. Any virus production induced in these resting T cell populations reflects the presence of latent HIV-1, because without T cell activation resting T cells are unable to generate virus particles [43]. The dilutions of cells are then stimulated with a T-cell activator such as the mitogen Phytohaemagglutinin (PHA) with irradiated allogeneic PBMCs or co-stimulatory antibodies, to induce virus production and reversal of latency. Subsequently, released virus is propagated by addition of cells susceptible to infection. This allows exponential expansion of virus produced from latently infected cell to the detection limit of viral antigen ELISA [38]. Though QVOA has been found to be sensitive and useful technique for estimating the outgrowth of replication competent virus, requirement of large volume of blood having low frequency of latently infected cells, labor intensive experimental set up and slower detection time restricts its scalability [42]. Moreover, due to the inadequate induction and/or propagation of replication competent virus, it underestimates the size of HIV reservoirs [44]. Therefore, alternative culture based methods estimating the inducible HIV reservoirs have been developed. In these assays, resting CD4^+^ T cells obtained from virally suppressed individuals are activated in presence of antiretrovirals; the cell associated-RNA from the cell extracts and cell free-RNA from cell supernatants are measured. However, the limitation is cell associated RNA detects defective genomes along with transcriptionally competent provirus. Likewise, measurement of cell free-RNA does not reveal the replication competency of viral particles [44].

Further advancements in PCR based techniques for quantifying the replication competent proviruses have become more reliable and feasible options. These includes, (**1**) Standard PCR that amplifies the short conserved regions of provirus, (**2**) Alu-PCR that amplifies the regions between Alu element and differentiating integrated and non-integrated proviruses, and (**3)** Digital droplet PCR (ddPCR) that distinguishes most deleted and/or hypermutated proviruses from the intact proviruses using two amplicons and hypermutation discrimination probes [45]. Subsequently, by using the intact proviral DNA assay (IPDA), intact and defective proviruses can be distinguished using plasmid controls that represent proviruses with multiple defects. It has been reported that IPDA is a promising approach for detecting the intact proviruses [45,46]. However, IPDA only amplifies the a fragment of HIV genome, but not whole HIV-LTR [47]. This limitation may reduce its applicability is accurate discrimination of intact and defective proviruses. Thus to resolve this, in-depth studies are needed that carries a promising options for measurement of reservoir size that ultimately affect monitoring the prognosis of HIV infection and the cure strategies.

Through these observations it can be summarized that standard QVOA underestimates the reservoir size whereas PCR based techniques may overestimate it. Alternatively, the methods estimating the inducible HIV reservoirs might provide clear idea on this issue.

### 2.5 Screening of latency reversing agents (LRAs)

Persistence of HIV-1 in both circulating and resting CD4^+^ T cells has highlighted the need for studying underlined mechanisms of HIV latency and its reversal using senotherapeutics. The senotherapeutics are the pharmacologic agents that target senescent cells, a step forward to combat HIV-1 persistence. Recently, it has been evidenced that chronic HIV-1 infection gives rise to accelerated aging and it is associated with senescence in circulating CD4^+^ T cells. Also, ART suppressed HIV-infected individuals have chronic immune activation, despite the elimination of AIDS related infections [45]. Moreover, inflammation has been found to be associated with increased risk of organ impairment and non-AIDS associated malignancies. Therefore, senotherapeutics, specifically the senolytics have been identified for suppression of senescent cells by targeting pathways essential for induction and maintenance of senescence. Among various strategies, “shock and kill” strategy based on the pharmacologic intervention using LRAs has been studied in detail.

The FDA approved drug, Panobinostat treating multiple myeloma, shown senolytic properties, has demonstrated the anti-reservoir effect [45]. Another CCR5 antagonist Maraviroc (MVC) has also been identified as latency reversing agent that induces HIV-1 production [48]. The latency reversal potential of MVC was evaluated in three cell line latency models and in *ex vivo* CD4^+^ T cells from patient samples with suppressive therapy. Using TZM-bl cells and by measuring HIV-1-LTR-driven luciferase gene expression HIV induction was evaluated. Whereas, by using latently infected ACH-2 and U1 cell lines, cell free and cell associated HIV-1 RNA was quantified by qPCR. However, the effects of MVC has shown to be cell line specific that depends on CCR5 expression and nature of viral latency. Therefore, the study findings revealed from cell line specific model cannot be generalized and are not directly applied to *in vivo* settings. Though the study underlines the utility of cell line based systems for screening of candidate LRAs, however, suggesting more investigation on development of *in vivo* models for disrupting HIV-1 latency.

### 2.6 RNA based therapeutics

In the recent years, RNA based therapeutics and delivery systems have become promising and achieveable approaches for therapeutic interventions against many dieseases [3]. In case of HIV infection a controlled HIV-1 replication upon stopping drug therapy have been explored to achieve the functional cure. Among them, HAART has been the most promising approach, though not without the challenges like drug resistance, latent reservoirs etc. Therefore, mining of novel antiviral agents and their innovative delivery systems are in continuous demand. RNA therapeutics, cell and gene therapies have been innovative and promising but evolving therapeutic options to combat HIV infection [17]. Together these efforts may lead to the HIV-functional cure in the following years.

RNA therapeutics and their delivery vehicles are targeted at the desired cells. There, they can manipulate or suppress gene expression, even produce a therapeutic proteins. These properties have attracted attention of researches for using them as pharmaceuticals. Main types of RNA therapeutics are based on antisense oligonucleotides (ASOs), small interfering RNAs (siRNAs), and messenger RNA (mRNA). ASOs and siRNAs utilize RNAse H1 and RNA-induced silencing complex (RISC), the enzymes endogenously produced by eukaryotic cells [49]. ASOs and siRNAs both bind to mRNAs through base pairing and by recruiting their respective enzymes, modulate translation, splicing and lead to cleavage of the target RNA. ASOs also interfere with pre-mRNA whereas siRNAs act by silencing the target genes and by reducing the expression of protein-coding gene. Interestingly, siRNAs have opened an option to combat HIV-1 by tailoring their sequence to patient specific viral strain, leading to personalized therapy for HIV-1 infection [50]. However, lymphocytes pose the major challenge towards development of *anti*-HIV-1 siRNAs due to their wide distribution in the body and difficulties in penetration using available siRNA delivery technologies [51]. These obstacles demands more studies on this topic so that these siRNA based therapeutics will also have longer shelf life, oral administration and high efficacy with low dosage frequency.

The therapeutics based on mRNA are complementary to the DNA strand of a gene and do not get integrated into the genome that makes them suitable for vaccines and tumor immunotherapy [52,53]. Also, the mRNAs, after reaching the cytoplasm are translated into proteins which do not require nuclear entry to be functional [54]. Further, mRNA can also be used to replace proteins, reduce protein levels, and repair DNA-level protein mutation [[55], [56], [57]]. However, there are many challenges for mRNAs turned into the drugs. Significant challenges have been identified as instability, enzymatic degradation, undesirable immune response and large-scale production [53]. Moreover, as RNA has no intrinsic targeting abilities, it needs directed delivery requiring some vehicle or modifications to gain entry into the desired cell [3]. To date, the novel delivery methods for RNA therapeutics that have been studied are microinjection, gene gun, RNA adjuvants, sugar or peptide conjugates and nano-liposomes. Although, diverse methodologies have evolved for improved delivery system, extensive research in this area is still in surge. Eventually, current available experimental strategies to evaluate RNA therapeutics shall also be progressed.

Summarizing the approach, it can be stated that RNA based therapeutics exhibit a range of MoAs including modulation or silencing of gene and protein expression or suppression. Moreover, the main advantage is, RNA based drugs are simple to plan because their design is based on base-pairing interactions [55]. Therefore, once the sequence of target molecule is known it is easy to design a RNA based therapeutics that bear the potential to combat many infectious and noninfectious diseases.

### 2.7 Applications of 3D cell culture system in antiretroviral drug discovery

Two dimensional (2D) cell-based assays have furnished reliable, reproducible, and cost-effective methods for identifying novel drugs, since years. Still, these monolayer cultures are not free of limitations. They need plastic substrates to grow and unlike *in vivo* environment*,* lack three-dimensional (3D) architecture. Furthermore, 2D and 3D cell culture systems differ in cellular responses at the following aspects: 1. Physical and physiological properties like, morphological, nutritional & functional; 2. The spatial organization of surface receptors and binding efficiency of drugs to these receptors; 3. Gene, protein and cell receptor expression; 4. Variation in cell stages, 2D cultured cells are primarily in the proliferating stage, while 3D culture is a mixture of heterogeneous cell populations that propagate at different stages. Thus only 3D cell cultures can mimic *in vivo* cellular heterogeneity, morphology and function, cell-cell interaction and diffusion barriers. Furthermore, it has also been reported that, 2D cell culture system sometime provides misleading and non-predictive data for *in vivo* responses, leading to misfortune of many drugs during the most expensive phase III clinical trials [[58], [59], [60]]. Aforesaid misfortunes have outstretched the need for new preclinical models that can reiterate the *in vivo* biology and microenvironment in a cell. As a result, 3D cell culture systems have become progressive and feasible in the field of drug discovery and development. Further, *in vitro* toxicity screens conducted on 3D cell culture models have demonstrated the three influencing factors on drug responsiveness. These include, particular cell line, tumor type in case of cancers, and its surrounding stroma [[61], [62], [63]]. Similarly, it has been observed that, the therapeutic efficacy, drug resistance or enhanced sensitivity of drug candidates may vary with tissue-specific composition of the ECM, the interaction with stromal cells and immunomodulatory molecules [[61], [62], [63]].

Broadly, in 3D cell culture, cells are grown into spheroids or aggregates using cellular scaffolds and matrices, providing physical support required for cells to attach, grow and migrate. These scaffolds are of two types, biologically derived and synthetic. Hyaluronic acid and basement membrane matrix (Matrigel) are commonly used biologically matrices. Amongst the synthetic scaffolds, Polyethylene glycol (PEG) and polyvinyl alcohol (PVA) are common in use [60]. The 3D environment can also be created by culturing cells in a scaffold-free manner, wherein cells suspensions are generated by forced floating method, hanging drop method, or agitation-based techniques [60]. Using any of these methods, the cells are allowed to grow naturally in their 3D-microenvironment, with sufficient interaction between the cells and ECM. As a result, the 3D spatial organization of cells influences cellular functions such as proliferation, differentiation, morphology, gene, protein and receptor expression, and cellular responses to external stimuli. All these merits have nominated 3D cell culture system as a bridging gap between 2D-cell-based assays and animal studies. In addition, it has been assured that, this strategy would generate more relevant and predictive data compared to conventional methods [64].

3D cell culture model also holds a promise for understanding the fundamentals of HIV-1 pathogenesis and infection dynamics because of its tissue-like fabrication that mimics the *in vivo* physiology [65]. Recently, three-dimensional human brain orgonoid model (hBORG) has been developed to study HIV–CNS pathology and the underlying molecular events of HIV-1 associated neurocognitive disorder (HAND) [65]. Briefly, following systemic-HIV-1 infection, regardless of suppressive ART, the virus infiltrates through the blood brain barrier, infect microglia and other glial cells. Consequently, dysfunctioning glial cells and neuroinflammation leads to neurodegeneration, resulting in cognitive impairment in 50% of HIV infected individuals. This brain-mimicking hBORG model has been developed with major cell types important for HIV-1 associated neuropathogenesis involving infected and uninfected microglia, neurons and astrocytes [65]. Generating this multicellular network and representing HIV infected human brain, chronic neuroinflammatory environment has been studied in HIV-infected individuals. The model has offered basic knowledge about alteration of CNS compartment upon HIV infection and consequent pathological changes directing the field towards discovery of biomarkers and new therapeutic agents.

Similarly, 3D organoid models, representing specific organ or tissue, have been developed to study HIV-1 disease pathology, biomarkers and to evaluate therapeutic interventions. The main goal of antiretroviral research is to identify safe, effective ART regimens and/or preventive therapeutic interventions for the entire population of HIV positive and at-risk individuals. Additionally, an adverse effect of long-term administration of ART needs attention. 3D cell culture models offer complementary information that help in bridging the gap between preclinical studies and clinical observational studies [66,67].

The development of microbicide, a multifunctional chitosan-lipid nanocomplexes that can effectively deliver plasmids encoding siRNA(s) have been evaluated using 3-dimensional human vaginal ectocervical tissue (3D-VEC). The study demonstrated that chitosan-lipid (chlipid), complexed with a cocktail of plasmids encoding HIV-1-specific siRNAs (psiRNAs) has provided significant protection against HIV in both *in vitro* and *in vivo* models, without any adverse effects [66]. Recently, newer approaches including human induced pluripotent stem cell (iPSC)-based 3D cell culture models have also been developed to study the impact of ART on fetal, postnatal neurodevelopment and also for predicting safety of ART drugs during pregnancy [67]. Of late, a region-specific model of forebrain, cortical organoid has also been fabricated wherein specific complement of patterning factors leading to the self-organized formation of ventricular zones with neurons expressing the markers of all cortical layers and the basic laminar structure of the human cortex has been successfully developed [68]. Although, transmission modes of HIV-1, viz. cell-free, cell associated and cell-cell transmission are known, its spread in the *in vivo* conditions is still elusive.

### 2.8 3D models and HIV-1 cell-cell transmission studies

HIV spread between CD4^+^ T lymphocytes and reduced motility of CD4^+^ T cells following HIV infection has been studied by 3D cell culture system [69]. The 3D cell culture system has been established using the collagen scaffold and HIV-1 infected primary human CD4^+^ T lymphocytes. The 3D cell culture system has facilitated the study of various factors such as pathogen infectivity and diffusion, replication dynamics, cell motility and cell-cell interaction that could not be possible with 2D cell culture system. The 3D model has reciprocated the motility of CD4^+^ T cells reduces upon HIV-1 infection [69]. Subsequently, the study has also revealed that a 3D environment promotes the cell-to-cell transmission of HIV but suppresses the transmission of cell-free viral particles.

Central mechanism of HIV transmission is production of infectious virus particles that target bystander cells. Cell-associated transmission occurs by short-distanced spread of cell free virus at cell-cell contacts, through cell protrusions that connect donor and target cells and also by cell-cell fusion. Out of these transmission modes, cell associated mode is considered more efficient than cell free infections [69]. Moreover, a structure called virological synapse, comprising the polarized delivery of newly formed virus particles into the target cells has been an important mechanism of transmission. This requires both cellular and viral proteins for the spread through nanotubes, filopodia and phagocytic compartments [70]. Though, much is known about how HIV uses cell associated modes of transmission in the experimental set up, its mode of transmission and how specific tissue-environment affects its spread in *in vivo* system, is yet to be revealed. Therefore, to reflect the complex tissue environment and host interactions with virus *in vivo* infection models would be the best option. Considering the limitations of 2D cell culture systems and organoid cultures, such as cell density and composition, donor-variability and limited availability of tissues, 3D cell culture systems would match the experimental requirements [69]. Particularly for lymphocytes and dendritic cells, collagen based 3D matrices would truly reflect specific aspects of tissue organization and physiology.

Furthermore, 3D cell culture model has given an opportunity to study HIV-HSV-2 co-infection [71]. For this, organotypic epithelial raft cultures of primary human keratinocytes (PHKs), has been developed for evaluating the suppression of transmission and replication of both the viruses by multi-targeted drug candidates. The collagen rafts have been used to set up organotypic raft cultures of PHKs. The study has revealed that, the dual-targeted antiviral drug has suppressed HSV-2-DNA polymerase, HIV-1-reverse transcriptase and most importantly, it can stimulate secretion of CC-chemokines that downregulate CCR5 of HIV-1 [71]. This downregulation of CCR5 co-receptors has demonstrated the indirect immunomodulatory effect of the investigated novel drug. Hence, by mimicking the disease conditions, 3D cell cultures would eventually deliver more knowledge about unrevealed drug targets. In addition, they can encourage the studies on dual targeting drug molecules and their immunomodulatory effects as well. 3D cell system thus generated by selecting appropriate cell population, allowing co-infection of two or even more number of viruses, would provide more realistic and reliable knowledge on viral interactions, their pathogenicity and effects of their replication on each other. Eventually strategies for therapeutic and clinical interventions may be planned on the basis of this knowledge.

In spite of these merits, the 3D cell culture system has presented some important challenges. To name the few, requirement of costly material, scalability, reproducibility and most importantly labour intensiveness, pointing the limited use of HTS platform [64,72]. Moreover, the natural source of collagen and matrigel has also found to be the limiting factor because batch variation in their preparation affects reproducibility in results. Still, with the help of innovative, customizable and ready-made scaffold systems, 3D cell culture has always paved a progressive path towards the creation of disease model enabling better understanding of diverse pathogenic mechanisms caused by wide range of etiological agents. Consequently, such realistic data generated from these *in vivo-like* conditions, at early preclinical step, would definitely contribute in accelerating the process of drug design and development.

## 3 Conclusion

In the past few decades it has been demonstrated that the cell based strategies have contributed massively in the antiretroviral drug development campaigns. Additional efforts in advancements of therapeutic interventions, have presented better pharmaceutical options and their delivery systems. Consequently, for evaluation of these drug-like agents, wide range of screening platforms has evolved. With this review, we have made an effort to outline the importance and feasibility of current cell based methodologies required for determining the saftey and efficacy of candidate antiretrovirals. The platforms facilitating the assesment of cellullar viability, toxicity and efficacy of drug candidates, showcase the preliminary understanding of drug's destiny in the upcoming clinical trials. As yet several studies have shown that, precise characterization and accurate determination of mechanism of action of drug candidtaes at early-preclinical stage will be helpful for future directions, shortening the timespan of clinical trials. Moreover, the traditional cell based platforms supported by innovative and reliable spectroscopic and in silico techniques will strengthen the drug mining drives. However, several novel but less revealed approaches like latent reservoir quantification, RNA based therapeutics, nanoparticle based delivery systems and 3D cell culture techniques need more attention with respect to their validity and reproducibility while screening antiretrovirals. Furthermore, their substitution or conjugation with traditional cell based systems would be benificial for defeating the obstacles imposed by the conventional systems. For example, the 2D cell culture systems can be substituted by 3D cell culture platforms for better understanding of the disease pathology, thereby generating more realistic data on safety and efficacy of drug-like molecules. Further, an incorporation of automoated platforms and miniaturization in all the plausible experiments will facilitate screening of large number of antiretroviral agents at a time in an error-free fashion, therefore reducing number of years of clinical trials and improving the final outcome.

## Author contribution statement

All authors listed have significantly contributed to the development and the writing of this article.

## Data availability statement

No data was used for the research described in the article.

## Declaration of competing interest

The authors declare that they have no known competing financial interests or personal relationships that could have appeared to influence the work reported in this paper.
